# Quality of life of patients with coronary heart disease treated with the bioresorbable vascular scaffold (ABSORB™): 2-year results from the GABI-R-registry

**DOI:** 10.1186/s12872-022-02815-2

**Published:** 2022-08-20

**Authors:** Kathrin Pahmeier, Silke Neusser, Christian Hamm, Johannes Kastner, Jochen Wöhrle, Ralf Zahn, Stephan Achenbach, Julinda Mehilli, Tommaso Gori, Christoph Naber, Holger Nef, Till Neumann, Gert Richardt, Axel Schmermund, Christoph Claas, Thomas Riemer, Janine Biermann-Stallwitz, For the GABI-R Study Group

**Affiliations:** 1 Institute for Health Care Management and Research, University Duisburg-Essen, Thea-Leymann-Str. 9, 45127 Essen, Germany; 2 University of Giessen, Medical Clinic I, 35392 Giessen, Germany; 3 Division of Cardiology, Department of Medicine II, Medical University of Vienna, 1090 Vienna, Austria; 4 Department of Cardiology, Medical Campus Lake Constance, 88048 Friedrichshafen, Germany; 5 Department of Cardiology, Clinical Center of the City of Ludwigshafen, 67063 Ludwigshafen, Germany; 6 Friedrich-Alexander-Universität Erlangen-Nürnberg (FAU), Medizinische Klinik 2, 91054 Erlangen, Germany; 7 Department of Internal Medicine I (Cardiology), Ludwig-Maximilians-University of Munich, 81377 Munich, Germany; 8 Department of Cardiology I, University Medical Center, Johannes Gutenberg University Mainz, 55131 Mainz, Germany; 9 DZHK Standort Rhein Main, Rhine-Main, Germany; 10 Department of Internal Medicine I, Cardiology and Intensive Care, Klinikum Wilhelmshaven, 26389 Wilhelmshaven, Germany; 11 Outpatient Department Cardiology, 44866 Bochum-Wattenscheid, Germany; 12 Department of Cardiology and Angiology, Segeberger Kliniken GmbH, 23795 Bad Segeberg, Germany; 13 Cardioangiologisches Centrum Bethanien, CCB, 60389 Frankfurt am Main, Germany; 14 IHF GmbH, Institut Für Herzinfarktforschung, 67063 Ludwigshafen, Germany

**Keywords:** Coronary artery disease, Quality of life, Stents, Bioresorbable vascular scaffold

## Abstract

**Background:**

Numerous studies have reported clinical endpoints following coronary revascularization using bioresorbable vascular scaffolds (BVS), while information about the impact on health-related quality of life is sparse. In this analysis of the German-Austrian ABSORB RegIstRy, the 2 year results concerning quality of life development in a large cohort of patients treated with BVS were reported.

**Methods:**

Data were collected at baseline as well as 30 days, 6 and 24 months after coronary revascularization using BVS. The EQ-5D score, EQ visual analogue scale (VAS) and Seattle Angina Questionnaire (SAQ) were determined for each time point. Patients were categorized according to the indication for coronary revascularization [acute coronary syndrome (ACS), stable angina pectoris (SAP), silent myocardial ischemia (SMI), or other]. Binary logistic regression analysis was performed to determine factors that predict above-average scores two years after implantation.

**Results:**

Data from 1317 patients in 88 centres were included. Reasons for revascularization were: ACS (n = 643), SAP (n = 443), SMI (n = 52), and other (n = 179). Mean EQ-5D was significantly increased after six months, while a value comparable to baseline was found two years after implantation. EQ VAS and four of five dimensions of SAQ were significantly improved over baseline at all follow-up surveys. Particularly strong improvements were seen in SAQ scores angina frequency and quality of life. Binary regressions showed different statistically significant predictors in the respective models.

**Conclusions:**

Following coronary revascularization with BVS strong decrease in self-reported angina frequency and increase of self-reported quality of life were observed with continuous improvements over two years of follow-up.

*Trial registration* ClinicalTrials.gov Identifier: NCT02066623.

**Supplementary Information:**

The online version contains supplementary material available at 10.1186/s12872-022-02815-2.

## Background

Patients with coronary heart disease (CHD) have substantial physical and mental problems as well as impaired health-related quality of life [1, 2]. Studies highlighted that CHD may result in loss of ability to work, thereby leading to disability, early retirement and elevated risk of cardiovascular mortality [3–5]. Furthermore, CHD is often associated with depression which considerably impairs quality of life and impose a great financial burden on society [6].

Coronary stents are the most frequently used devices for percutaneous coronary intervention in the treatment of patients with CHD [7]. In addition to the conventional drug-eluting or bare metal stent, which remain permanently in the vessel, bioresorbable vascular scaffolds (BVS), which dissolve within a few years after implantation, are one of the latest innovations [8]. Clinical trials showed very late device thrombosis associated with the ABSORB BVS [9] as well as higher rates of target lesion failure and target vessel-related myocardial infarction compared to an everolimus-eluting metallic stent [10]. Due to low commercial sales Abbott stopped selling the 1st generation ABSORB™ BVS.

Numerous studies have reported clinical endpoints (e.g. mortality, scaffold thrombosis, target lesion failure), while information about quality of life after BVS implantation is sparse. In a single-blind, multicenter, randomized trial (ABSORB II), Serruys et al. examined the angina status assessed by the Seattle Angina Questionnaire (SAQ) in addition to primary clinical endpoints. Patients were randomly assigned to a treatment with BVS or with an everolimus-eluting metallic stent. 6 and 12 months after coronary interventions, the five domains of SAQ were improved in both groups compared to the pre-procedural assessment. No statistically significant difference could be determined between the treatment groups [11]. In another multicenter study, patients treated with BVS showed significantly better results in four of five SAQ dimensions two years after implantation as compared to those who had received a metallic stent [12].

The present analysis reports the 2 year results of the German-Austrian ABSORB RegIstRy (GABI-R) on the quality of life development of a large cohort of patients treated with BVS.

## Methods

GABI-R is a prospective, multicenter, non-interventional, observational study of patients who have been treated with the ABSORB™ BVS due to coronary artery stenosis. The follow-up period is at least 5 years. Patients had to sign an informed consent form for inclusion. The study considered the Declaration of Helsinki and was approved by the ethics board of the University of Giessen [13].

Data on health-related quality of life were collected at baseline in the clinical setting as well as 30 days, 6 months and 24 months after stent implantation in telephone interviews. Detailed information on the questionnaire can be found in the publication by Nef et al. [13]. Quality of life was measured with the EQ-5D-5L including visual analogue scale (VAS) and the disease-specific instrument SAQ. The official translated versions of both instruments were under license. Therefore, licenses for the EQ-5D-5L and the SAQ were obtained. To determine quality of life development over time patients were included in the analysis (1) if they were alive and provided complete information on quality of life (EQ-5D-5L, VAS, SAQ) at baseline and all follow-up surveys, (2) if they had died between the implantation and one of the follow-up surveys or (3) if they died shortly after implantation. In the latter two cases patients were included if they had previously provided complete information on quality of life.

Statistical analysis was performed with SAS^®^ 9.4. To calculate EQ-5D scores, the German reference values by Ludwig et al. [14] were used. Mean values and standard deviations were determined for each time of survey. Values of the follow-up surveys were tested for statistically significant differences compared to the baseline value using a paired sample t-test.

A sensitivity analysis was performed to determine the influence of the deceased on the development of quality of life. Only patients who had not died during the follow-up period of 2 years were included in this analysis.

Analogous to the analysis of the entire study population, quality of life of the included patients was analysed according to their indication: acute coronary syndrome (ACS), stable angina pectoris (SAP), silent myocardial ischemia (SMI) and other indications. In the last group, patients with an undetermined diagnosis or a mixed form from one of the three main diagnoses (e.g. ACS and SAP) were summarised.

To determine factors that influence an above average quality of life two years after implantation, binary logistic regressions were used, one for each score. The dependent variable was defined as exceeding the average score. Exogenous variables included indication, scores of EQ-5D, EQ VAS and SAQ dimensions at baseline, health care costs at baseline, 6 months and 24 months after implantation, medication, various comorbidities, family history of CHD, previous interventions before stent implantation at baseline, condition after resuscitation and some personal characteristics like age, gender and type of health insurance. An overview of all variables used is given in the supplement. The significance level was set at 5%.

## Results

Data from 1317 patients in 88 centres were included in the analysis of health-related quality of life. Patients had an average age of 61 years and the majority were men (80%; Table 1). Approximately one half of the study population were former or habitual smokers. 643 patients suffered from ACS, 443 from SAP, 52 from SMI and 179 patients had other indications or a mixed form. Of the 643 patients with ACS, 278 had non-ST-elevation myocardial infarction, 216 ST-elevation myocardial infarction and 149 unstable angina.

**Table 1:** Patient characteristics at baseline

	N	Study population (n = 1317) [n (%)]
Age in years [mean ± SD]		61.2 ± 10.5
Male sex		1048 (79.6)
Smoker (former or habitual)	1245	706 (53.6)
*Indication*		
Acute coronary syndrome		643 (48.8)
Stable angina pectoris		443 (33.6)
Silent myocardial ischemia		52 (3.9)
Other		179 (13.6)
*Comorbidities*		
Atrial fibrillation	1294	86 (6.5)
Arterial hypertension	1303	950 (72.1)
Carotid arterial disease	1317	39 (3.0)
Cancer	1317	58 (4.4)
COPD	1317	67 (5.1)
Diabetes mellitus type II	1301	219 (16.6)
Hyperlipoproteinemia	1254	700 (53.2)
Pulmonary artery pressure	1317	55 (4.2)
Renal failure	1308	90 (6.8)
Stroke	1317	29 (2.2)
Thyroid dysfunction	1317	118 (9.0)
Transient ischemic attack	1317	10 (0.8)
*Prior coronary treatment or examination*		
Coronary angiography	1299	450 (34.2)
CABG	1313	31 (2.4)
PCI without stenting	1022	103 (9.8)
PCI with stenting	1293	363 (27.6)
Bare metal stent	298	33 (2.6)
Drug eluting stent	305	231 (17.8)
Bioresorbable vascular scaffold	300	64 (5.0)
Implant (e.g. pacemaker)	832	22 (2.6)

*N* Patients with non-missing values. *SD* Standard deviation

*CABG* Coronary artery bypass grafting; *COPD* Chronic obstructive pulmonary disease; *PCI* Percutaneous coronary intervention

The most common comorbidities were arterial hypertension (72%) and hyperlipoproteinemia (53%). 17% of patients reported suffering from diabetes mellitus type II. About a third of the study population had a coronary angiography before. 28% of patients reported a prior percutaneous coronary intervention with stenting. In total, 46 patients (3%) died between baseline and 24-months-follow-up. 10 patients died within one month of the intervention (5 with ACS, 2 with SAP, 2 with SMI and 1 with other indication), of which seven died during the index hospitalization.

Table 2 lists the mean values and standard deviations of EQ-5D score, EQ VAS and SAQ scores of the entire study population for each time of survey. The EQ-5D score increased statistically significantly 30 days after stent implantation (baseline: 0.88 ± 0.19; 30 days-FU: 0.91 ± 0.15; *p* < 0.0001). In the further course the EQ-5D score decreased. Six months after implantation the EQ-5D score was statistically significantly different compared to baseline (0.90 ± 0.16; *p* = 0.0009). Two years after implantation, the score reached a level comparable to baseline (0.89 ± 0.2; *p* = 0.6697).

**Fig. 1:**
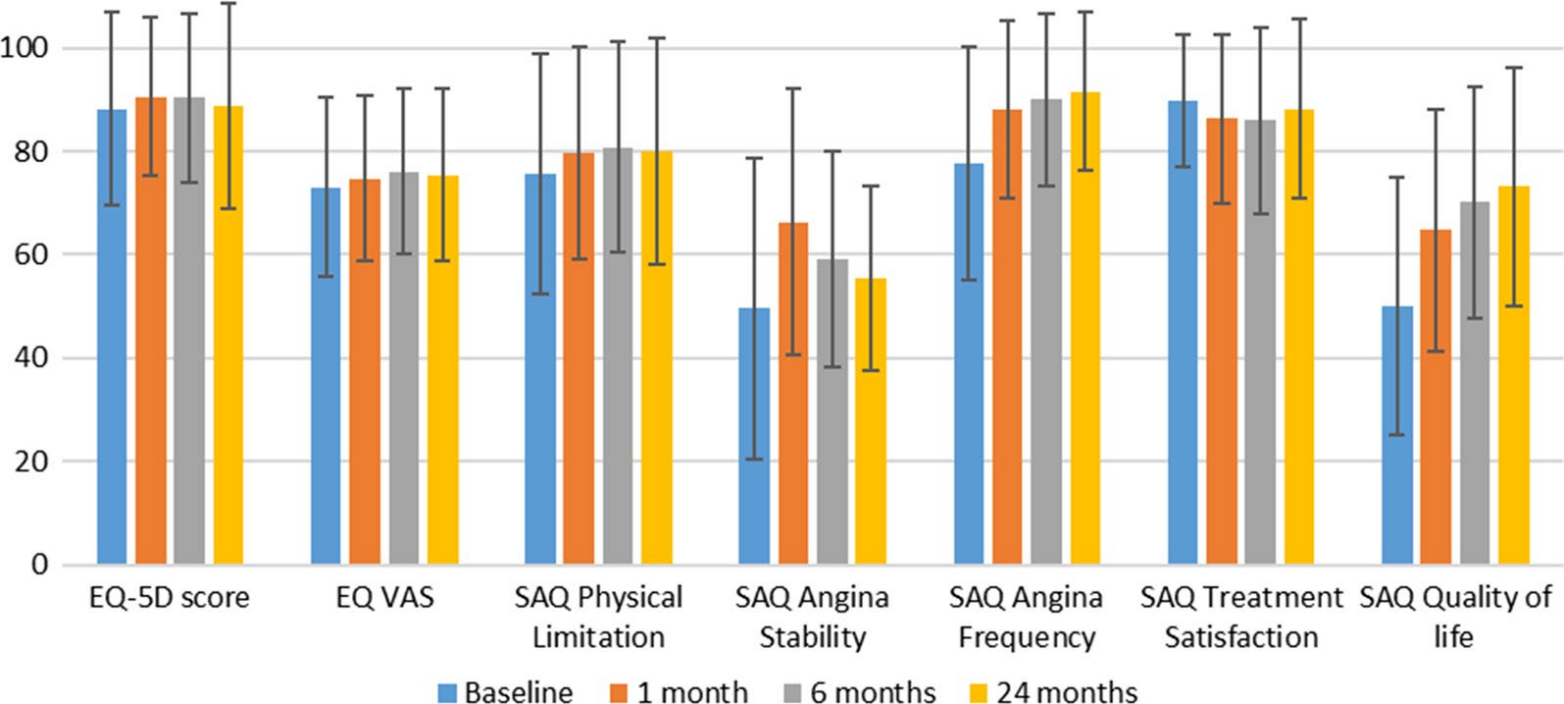
Quality of life values of the entire study population. To use a uniform labelling of the y-axis across all scales, the values of the EQ-5D score were multiplied by 100

EQ VAS and the five dimensions of the SAQ were statistically significantly different at all follow-up surveys compared to the respective baseline value. With exception of the SAQ dimension treatment satisfaction, the average values increased at the first follow-up point (30 days after implantation), which corresponds to an improvement in health-related quality of life. EQ VAS and the SAQ dimension physical limitation continued to increase for the 6-month follow-up, while the average value of the SAQ dimension angina stability decreased. Two years after implantation the EQ-VAS and the SAQ dimensions physical limitation and angina stability decreased on average compared to the previous follow-up survey, but were statistically significantly higher compared to baseline. Regarding the SAQ dimensions angina frequency and quality of life, there were significant improvements 30 days after coronary intervention. The average values of the angina frequency and quality of life scores increased from 77.6 ± 22.6 to 88.1 ± 17.2 (*p* < 0.0001) and 50.0 ± 24.8 to 64.8 ± 23.4 (*p* < 0.0001), respectively. This development continued 6 months (angina frequency: 90.0 ± 16.6, *p* < 0.0001; quality of life: 70.1 ± 22.4, *p* < 0.0001) and 2 years (angina frequency: 91.6 ± 15.3, *p* < 0.0001; quality of life: 73.1 ± 23.0, *p* < 0.0001) after the intervention (Fig. 1).

**Table 2:** Quality of life values of the entire study population

	Entire study population (n = 1317)			
Scale	Baseline (n = 1317)	30 days (n = 1307)	6 months (n = 1298)	24 months (n = 1290)
EQ-5D score	0.88 (0.19)	0.91 (0.15)*	0.90 (0.16)*	0.89 (0.20)
EQ VAS	73.0 (17.3)	74.7 (15.9)*	76.1 (16.1)*	75.4 (16.6)*
SAQ Physical Limitation	75.5 (23.2)	79.6 (20.5)*	80.8 (20.3)*	80.0 (21.8)*
SAQ Angina Stability	49.6 (29.0)	66.3 (25.6)*	59.1 (20.8)*	55.4 (18.0)*
SAQ Angina Frequency	77.6 (22.6)	88.1 (17.2)*	90.0 (16.6)*	91.6 (15.3)*
SAQ Treatment Satisfaction	89.8 (12.8)	86.2 (16.5)*	86.0 (18.1)*	88.1 (17.3)*
SAQ Quality of life	50.0 (24.8)	64.8 (23.4)*	70.1 (22.4)*	73.1 (23.0)*

Falling sample size due to deaths

Mean values (standard deviation)

*SAQ* Seattle angina questionnaire; *VAS* Visual analogue scale

*: *p* < 0.05 (paired sample t-test in comparison to baseline)

In a sensitivity analysis the quality of life of patients who did not die during the course of the study was examined. Average values of most scores did not change or increased slightly. Compared to the analysis of the entire study population, the EQ-5D score after 24 months (0.90 ± 0.17) was significantly higher than at baseline (0.88 ± 0.18; *p* = 0.0024). Similar to the development of the entire study population, the EQ-5D score decreased slightly over time. Average values of the SAQ dimension quality of life at baseline and 30 days after the intervention were slightly lower compared to the entire study population. Detailed results of the sensitivity analysis can be found in the supplement.

Patients with ACS had a statistically significantly larger average EQ-5D score at all three follow-up surveys after BVS implantation (Additional file 1: Table S2). The average EQ-5D score and the average EQ VAS initially increased significantly in patients with SAP. Two years after implantation, the average values decreased to a level comparable to baseline. In SMI patients, there were mostly no significant changes in the EQ-5D score and EQ VAS compared to baseline at all follow-up times. The only exception was the average EQ-5D score 2 years after implantation, which was statistically significantly lower compared to baseline (*p* = 0.0437).

Patients with ACS and patients with SAP showed statistically significantly changed mean values in all SAQ scores at all follow-up times with one exception: the physical limitation score 30 days after BVS implantation of ACS patients was not statistically significantly higher (*p* = 0.0992). In contrast to the other four scores, which were consistently above the average baseline value, the SAQ treatment satisfaction score initially decreased in patients with ACS (*p* < 0.0001) and patients with SAP (*p* < 0.0001). Two years after BVS implantation, the average value was higher than at the previous follow-up surveys, but statistically significantly lower compared to baseline (ACS: *p* = 0.0205; SAP: *p* = 0.0030). Six (*p* = 0.0143) and 24 months (*p* = 0.0093) after stent implantation patients with SMI showed a statistically significantly higher SAQ angina frequency score compared to baseline and also a statistically significantly higher SAQ quality of life scale (*p* = 0.0007) 24 months after stent implantation. Detailed results are provided in Additional file 1.

To determine significant influence factors on achieving an above-average quality of life, several binary regressions were calculated, one regression for each score. None of the included variables was a significant predictor of all quality of life scores (Table 3). A higher EQ-5D score at baseline significantly increased the chance of achieving an above-average EQ-5D score, SAQ angina frequency score and SAQ quality of life score two years after implantation. An increase of the other baseline scores only slightly increased the chance of an above-average score. Higher health care costs after 6 or 24 months reduce the chance of achieving an above-average score, although the regression of the SAQ angina stability score was not statistically significant in this regard. These effects were so small (i.e. OR = 0.99992 in the first model) that the odds ratios were rounded to 1. Some comorbidities reduced the chance of an above-average score in some models, but no comorbidity was a significant factor in all models. The type of health insurance was a significant predictor for four of the scores mentioned. Patients insured by the statutory health system had a lower chance of achieving an above-average EQ-5D score, SAQ physical limitation score and SAQ quality of life score compared to patients with private insurance. In four models an older age reduced the chance of an above-average score. The indication for implantation had no significant influence in any model and is therefore not mentioned in Table 3.

**Table 3:** Significant influence factors (odds ratios) on achieving an above-average quality of life two years after implantation

	EQ-5D score	EQ VAS	SAQ physical limitation	SAQ angina stability	SAQ angina frequency	SAQ treatment satisfaction	SAQ quality of life
EQ-5D score baseline	7.964*** (3.73)	n. s	n. s	n. s	2.487* (1.03)	n. s	2.647* (1.13)
EQ VAS baseline	1.020*** (0.00)	1.032*** (0.00)	1.021*** (0.00)	n. s	1.009* (0.00)	1.010* (0.00)	1.009* (0.00)
SAQ physical limitation	1.012** (0.00)	1.012** (0.00)	1.027*** (0.00)	n. s	n. s	n. s	n. s
SAQ angina frequency	1.011** (0.00)	1.008* (0.00)	n. s	n. s	1.015*** (0.00)	1.009** (0.00)	n. s
SAQ treatment satisfaction	n. s	n. s	n. s	0.987* (0.01)	1.016** (0.01)	1.030*** (0.01)	1.022*** (0.01)
SAQ quality of life	n. s	n. s	n. s	n. s	n. s	n. s	1.011** (0.00)
Statutory health insurance	0.647* (0.13)	n. s	0.653* (0.12)	1.626* (0.35)	n. s	n. s	0.674* (0.11)
Age	0.982* (0.01)	0.970*** (0.01)	0.939*** (0.01)	0.981* (0.01)	n. s	n. s	n. s
Males	1.874** (0.34)	n. s	2.147*** (0.38)	n. s	1.498* (0.25)	n. s	1.648** (0.27)

Only statistically significant values are reported as odds ratios with standard errors in parentheses

*n. s.* not significant

* *p* < 0.05, ** *p* < 0.01, *** *p* < 0.001

## Discussion

This evaluation provides detailed descriptive information about the development of patients’ health-related quality of life and disease-specific health status after implantation of the ABSORB™ BVS. Within two years following implantation, the disease-specific quality of life measured with SAQ improved significantly over time in most dimensions, while the generic EQ-5D score decreased to a level comparable to baseline after an initial improvement. A possible explanation for these different evolvements could be the mainly elder and partly multimorbid population in this sample. The EQ-5D score includes dimensions that may be affected by other health problems. The SAQ as a disease-specific instrument, on the other hand, is more sensitive to changes that affect CHD in particular. This emphasizes the additional information gained through the use of disease-specific instruments for measuring quality of life.

Both the analysis of the entire study population and the analysis separated by indication for coronary intervention showed that significant and sustained improvements in the SAQ scores angina frequency and quality of life were achieved by implanting the BVS.

In patients with ACS and SAP similar trends are noticed, which, with the exception of the treatment satisfaction score of SAQ, mean statistically significant improvements compared to the initial situation. However, the baseline values differ between the indications. In most dimensions, patients with SAP had the lowest scores. This may be due to the chronic character of the disease with recurring complaints during physical activity or strong emotions. In a study by Manolis et al., 40% of 268 patients reported that the disease affects their quality of life despite treatment [15].

Serruys et al. examined the development of the disease-specific health status (measured with SAQ) of 335 patients treated with a BVS compared to 166 patients treated with an everolimus-eluting metallic stent (Xience, Abbott Vascular). SAQ was used before implantation as well as six months and one year after implantation. All dimensions of the SAQ showed an improvement after six months, which was still noticeable even one year after implantation [11]. This differs to our analysis, where the treatment satisfaction score was significantly lower at all follow-up surveys compared to baseline. With exception of the treatment satisfaction score, the mean values at baseline in the group of BVS treated patients are comparable to the values of the cohort included in our analysis. Treatment satisfaction score at baseline was on average 20 points lower than in our sample.

In the Spanish study, de la Torre Hernández et al. examined 208 patients undergoing percutaneous revascularization, 102 with BVS and 106 with drug-eluting stent. They report mean values and standard deviations of the five SAQ dimensions about two years after implantation [12]. The mean SAQ dimension quality of life (74.6 ± 23.0) was similar to our cohort. The means of the dimensions physical limitation (83.6 ± 23.7) and angina frequency (96.0 ± 8.0), and particularly the dimension angina stability (87.5 ± 21.8) were higher in the Spanish cohort. The dimension treatment satisfaction was lower on average (84.8 ± 18.2). However, due to a different focus of the Spanish study, no initial values are given, so that it cannot be discussed to what extent the patients’ quality of life has developed over time.

Regarding the SAQ dimension treatment satisfaction, which was significantly lower in our cohort after BVS implantation, other studies investigating quality of life after implantation of a drug eluting stent versus bypass surgery reported an improvement in this dimension after the intervention. [16, 17] However, the parameters that are included in the calculation of this dimension (e.g. satisfaction with physician’s explanations, satisfaction with treatment) do not necessarily indicate a connection with the device used.

The binary regressions showed different statistically significant influencing factors in the respective models, but none of the included variables was a significant predictor in each model. In three models, statutory health insured patients had a statistically significantly lower chance of achieving an above-average EQ-5D score or SAQ physical limitation score compared to those with private insurance, while in the SAQ angina stability model they had a higher chance of achieving an above-average score. In this regard, it should be noted that privately insured differ in several important parameters from that with statutory health insurance (e.g. income, years of education). These variables were not included in the data and so a potential influence of these variables could not be controlled. In a work by Hajek et al. (2018), the relevance of controlling for these parameters becomes apparent. The authors examined differences in morbidity by health insurance status in the elderly population. According to this, persons with statutory health insurance (SHI) have a higher morbidity than those with private health insurance. Men, increasing age and people with low education are also affected by a higher morbidity. Several regression models are estimated and by controlling for the income, the difference in morbidity between SHI and private health insurances decreases and is no longer significant in men [18].

The main limitation of the present study is the lack of a control group. Therefore, it cannot be determined what additional positive influence on quality of life the implantation of the BVS has compared to PCI with a bare metal or drug eluting stent. Nevertheless, the registry data from GABI-R make an important contribution to the development of quality of life over a period of two years after implantation due to the high number of 1317 patients whose data could be analysed. The results published by de la Torre Hernández et al. and described above are based on data from 102 patients treated with a BVS [12]. In the ABSORB II trial 335 patients treated with a BVS were enrolled [11].

The last assessment in GABI-R is carried out five years after implantation. With the evaluation of this data, further information about the long-term development of patients’ quality of life and disease-specific health status can be generated.

## Conclusions

Following coronary revascularization with BVS strong decrease in self-reported angina frequency and increase of self-reported quality of life were observed with continuous improvements over two years of follow-up. Even if no causal conclusion on the implantation of the BVS is permissible due to the registry data and the missing control group, the data show that the implantation of a BVS leads to a sustainable improvement in health-related quality of life.

## Supplementary Information

**Additional file 1.** Detailed results of the quality of life analysis.

## Data Availability

The datasets generated and analyzed during the current study are not publicly available and cannot be shared due to data protection. Data have been provided for the purpose of the study only.

## Abbreviations

ACS: Acute coronary syndrome
BVS: Bioresorbable vascular scaffolds
CHD: Coronary heart disease
COPD: Chronic obstructive pulmonary disease
CRT: Cardiac resynchronization therapy
GABI-R: German-Austrian ABSORB RegIstRy
PAD: Peripheral artery disease
PCI: Percutaneous coronary intervention
SAP: Stable angina pectoris
SAQ: Seattle angina questionnaire
SHI: Statutory health insurance
SMI: Silent myocardial ischemia
TIA: Transient ischemic attack
VAS: Visual analogue scale
