# Supplementary material for: Quality of life of patients with coronary heart disease treated with the bioresorbable vascular scaffold (ABSORB™): 2-year results from the GABI-R-registry
Source: BMC Cardiovasc Disord. 2022 Aug 20;22:379. doi: 10.1186/s12872-022-02815-2 (PMC9392935; doi:10.1186/s12872-022-02815-2)
Supplement: Supplementary file 1 — Additional file 1. Detailed results of the quality of life analysis. [file 12872_2022_2815_MOESM1_ESM.docx]

# Additional file 1: Detailed results of the quality of life analysis

Table S 1: Quality of life values: sensitivity analysis of the survivors

|  | **Sensitivity analysis of the survivors (n = 1,271)** | | | |
| --- | --- | --- | --- | --- |
| **Scale** | **Baseline** | **30 days** | **6 months** | **24 months** |
| EQ-5D score | 0.88 (0.18) | 0.91 (0.13)* | 0.91 (0.13)* | 0.90 (0.17)* |
| EQ VAS | 73.2 (17.3) | 74.8 (15.8)* | 76.1 (16.1)* | 75.4 (16.6)* |
| SAQ Physical Limitation | 75.8 (23.1) | 79.9 (20.4)* | 81.0 (20.2)* | 80.0 (21.8)* |
| SAQ Angina Stability | 49.7 (29.0) | 66.4 (25.7)* | 59.0 (20.8)* | 55.4 (18.0)* |
| SAQ Angina Frequency | 77.6 (22.6) | 88.1 (17.1)* | 90.0 (16.6)* | 91.6 (15.3)* |
| SAQ Treatment Satisfaction | 89.8 (12.8) | 86.1 (16.5)* | 85.9 (18.1)* | 88.1 (17.3)* |
| SAQ Quality of life | 49.8 (24.7) | 64.6 (23.3)* | 70.1 (22.4)* | 73.1 (23.0)* |
| Mean values (standard deviation).  *: p < 0.05 (paired sample t-test in comparison to baseline).  Falling sample size due to deaths.  SAQ: Seattle Angina Questionnaire; VAS: Visual analogue scale. | | | | |

Table S 2: Quality of life values by indication

| **Scale** | **Baseline** | **30 days** | **6 months** | **24 months** |
| --- | --- | --- | --- | --- |
| **ACS** | (n = 643) | (n = 638) | (n = 636) | (n = 633) |
| EQ-5D score | 0.89 (0.19) | 0.92 (0.14)* | 0.91 (0.14)* | 0.91 (0.15)* |
| EQ VAS | 73.7 (17.6) | 75.6 (15.1)* | 77.4 (15.5)* | 77.2 (15.3)* |
| Physical Limitation | 79.3 (22.8) | 80.9 (19.2) | 83.0 (18.6)* | 82.1 (20.1)* |
| Angina Stability | 48.1 (29.1) | 66.0 (25.8)* | 58.7 (20.2)* | 55.5 (16.8)* |
| Angina Frequency | 79.5 (22.2) | 88.9 (16.8)* | 90.5 (16.4)* | 92.9 (13.6)* |
| Treatment Satisfaction | 91.1 (11.4) | 86.9 (16.5)* | 86.9 (18.0)* | 89.6 (15.9)* |
| Quality of life | 49.9 (24.4) | 65.7 (22.6)* | 71.0 (22.0)* | 74.4 (21.8)* |
| **SAP** | (n = 443) | (n = 441) | (n = 437) | (n = 436) |
| EQ-5D score | 0.86 (0.20) | 0.89 (0.17)* | 0.89 (0.18)* | 0.86 (0.22) |
| EQ VAS | 71.4 (17.6) | 73.4 (16.4)* | 74.4 (17.2)* | 73.3 (17.6) |
| Physical Limitation | 68.9 (23.4) | 77.2 (22.3)* | 77.4 (22.0)* | 76.7 (23.7)* |
| Angina Stability | 49.5 (31.0) | 68.6 (26.1)* | 60.4 (22.4)* | 56.1 (20.7)* |
| Angina Frequency | 70.5 (23.2) | 86.0 (17.5)* | 88.1 (17.4)* | 89.0 (17.5)* |
| Treatment Satisfaction | 88.1 (14.0) | 84.8 (16.7)* | 83.8 (18.8)* | 85.1 (20.0)* |
| Quality of life | 45.7 (24.1) | 62.3 (24.1)* | 67.7 (23.0)* | 70.0 (24.4)* |
| **SMI** | (n = 52) | (n = 50) | (n = 50) | (n = 50) |
| EQ-5D score | 0.93 (0.14) | 0.93 (0.09) | 0.93 (0.11) | 0.86 (0.27)* |
| EQ VAS | 75.5 (16.4) | 76.1 (15.3) | 76.3 (16.2) | 76.3 (18.2) |
| Physical Limitation | 84.4 (16.1) | 83.3 (15.2) | 82.9 (19.1) | 83.2 (21.3) |
| Angina Stability | 58.2 (22.0) | 58.0 (22.8) | 54.5 (17.3) | 55.2 (14.5) |
| Angina Frequency | 87.9 (17.4) | 92.2 (12.8) | 94.6 (10.1)* | 95.6 (11.3)* |
| Treatment Satisfaction | 91.3 (11.6) | 89.7 (15.1) | 90.7 (11.7) | 93.2 (11.4) |
| Quality of life | 65.5 (23.1) | 68.2 (23.6) | 73.3 (21.3) | 80.5 (20.9)* |
| **Other** | (n = 179) | (n = 178) | (n = 175) | (n = 171) |
| EQ-5D score | 0.90 (0.15) | 0.90 (0.18) | 0.89 (0.20) | 0.87 (0.24) |
| EQ VAS | 73.5 (15.7) | 74.5 (21.7) | 75.7 (14.9) | 73.9 (17.7) |
| Physical Limitation | 76.1 (22.5) | 79.9 (21.7)* | 80.9 (21.3)* | 79.9 (22.4)* |
| Angina Stability | 52.8 (24.5) | 64.3 (23.6)* | 58.2 (19.5)* | 53.3 (15.5) |
| Angina Frequency | 85.2 (18.9) | 89.2 (18.5)* | 91.6 (16.7)* | 92.4 (15.7)* |
| Treatment Satisfaction | 88.9 (14.1) | 86.3 (15.7)* | 86.6 (17.4) | 88.8 (15.2) |
| Quality of life | 56.0 (25.8) | 66.8 (24.0)* | 72.0 (22.7)* | 74.1 (23.1)* |
| Mean values (standard deviation).  *: p < 0.05 (paired sample t-test in comparison to baseline).  Falling sample size within an indication due to deaths.  ACS: Acute coronary syndrome; SAP: Stable angina pectoris; SMI: Silent myocardial ischemia. | | | | |

## Factors potentially influencing an above average quality of life two years after implantation

As described in the methodology, we have used several variables to examine their influence on an above average quality of life two years after implantation. The following list shows all variables that were included in the model.

- Indication: acute coronary syndrome, stable angina, silent myocardial ischemia, (reference category: other indication)
- Scores of EQ-5D, EQ VAS and SAQ dimensions at baseline
- Health care costs at baseline, 6-months and 24 months after implantation
- Medication
- Comorbidities (respective reference category: not having the disease): hyperlipoproteinemia, kidney failure, diabetes mellitus type 1, diabetes mellitus type 2, chronic obstructive pulmonary disease (COPD), thyroid dysfunction, cancer, ventricular fibrillation, myocardial infarction, stroke, transient ischemic attack (TIA), peripheral artery disease (PAD), carotid arterial disease, other comorbidity
- Family history of CHD
- Previous interventions before stent implantation at baseline (respective reference category: not having the intervention): coronary angiography, bypass surgery, percutaneous coronary intervention (PCI) with stent, PCI without stent, cardiac operation, implants like pacemaker, defibrillator or cardiac resynchronization therapy (CRT)
- Condition after resuscitation
- Age
- Gender (reference category: female)
- Smoking status: smoker, former smoker (reference category: non-smoker)
- Type of health insurance: Statutory health insurance (reference category: private health insurance)
- Emergency admission (reference category: referral)

Table S 3: Significant influence factors (odds ratios) on achieving an above-average quality of life two years after implantation (complete regression results)

|  | **EQ-5D score** | **EQ VAS** | **SAQ Physical**  **Limitation** | **SAQ Angina**  **Stability** | **SAQ Angina**  **Frequency** | **SAQ Treatment**  **Satisfaction** | **SAQ Quality**  **of life** |
| --- | --- | --- | --- | --- | --- | --- | --- |
| EQ-5D score baseline | 7.964***  (3.73) | n. s. | n. s. | n. s. | 2.487*  (1.03) | n. s. | 2.647*  (1.13) |
| EQ VAS baseline | 1.020***  (0.00) | 1.032***  (0.00) | 1.021***  (0.00) | n. s. | 1.009*  (0.00) | 1.010*  (0.00) | 1.009*  (0.00) |
| SAQ Physical Limitation | 1.012**  (0.00) | 1.012**  (0.00) | 1.027***  (0.00) | n. s. | n. s. | n. s. | n. s. |
| SAQ Angina Frequency | 1.011**  (0.00) | 1.008*  (0.00) | n. s. | n. s. | 1.015***  (0.00) | 1.009**  (0.00) | n. s. |
| SAQ Treatment Satisfaction | n. s. | n. s. | n. s. | 0.987*  (0.01) | 1.016**  (0.01) | 1.030***  (0.01) | 1.022***  (0.01) |
| SAQ Quality of life | n. s. | n. s. | n. s. | n. s. | n. s. | n. s. | 1.011**  (0.00) |
| Health care costs after 6 months | 1.000***  (0.00) | 1.000**  (0.00) | 1.000***  (0.00) | n. s. | 1.000**  (0.00) | 1.000**  (0.00) | 1.000**  (0.00) |
| Health care costs after 24 months | 1.000***  (0.00) | 1.000***  (0.00) | 1.000***  (0.00) | n. s. | 1.000***  (0.00) | 1.000***  (0.00) | 1.000***  (0.00) |
| Diabetes mellitus type 1 | n. s. | n. s. | n. s. | n. s. | n. s. | n. s. | 0.191*  (0.15) |
| Diabetes mellitus type 2 | n. s. | 0.541**  (0.10) | n. s. | n. s. | n. s. | n. s. | n. s. |
| COPD | 0.378*  (0.15) | 0.333**  (0.13) | n. s. | n. s. | n. s. | n. s. | n. s. |
| TIA | n. s. | n. s. | n. s. | n. s. | n. s. | n. s. | 0.058*  (0.07) |
| Carotid arterial disease | 0.376*  (0.17) | n. s. | n. s. | n. s. | n. s. | n. s. | n. s. |
| Previous coronary angiography | n. s. | 0.596*  (0.14) | n. s. | n. s. | n. s. | n. s. | n. s. |
| Implants | n. s. | n. s. | n. s. | 2.973*  (1.55) | n. s. | n. s. | n. s. |
| Condition after resuscitation | n. s. | 3.065*  (1.68) | 3.260*  (1.78) | n. s. | n. s. | n. s. | n. s. |
| Smoker | n. s. | n. s. | 0.601**  (0.11) | n. s. | n. s. | n. s. | n. s. |
| Statutory health insurance | 0.647*  (0.13) | n. s. | 0.653*  (0.12) | 1.626*  (0.35) | n. s. | n. s. | 0.674*  (0.11) |
| Age | 0.982*  (0.01) | 0.970***  (0.01) | 0.939***  (0.01) | 0.981*  (0.01) | n. s. | n. s. | n. s. |
| Males | 1.874**  (0.34) | n. s. | 2.147***  (0.38) | n. s. | 1.498*  (0.25) | n. s. | 1.648**  (0.27) |
| * p<0.05, ** p<0.01, *** p<0.001.  Only statistically significant values are reported as odds ratios with standard errors in parentheses.  n. s.: not significant; ref.: reference category. | | | | | | | |
